# Vimentin expression is retained in erythroid cells differentiated from human iPSC and ESC and indicates dysregulation in these cells early in differentiation

**DOI:** 10.1186/s13287-019-1231-z

**Published:** 2019-04-29

**Authors:** Kongtana Trakarnsanga, Daniel Ferguson, Deborah E. Daniels, Rebecca E. Griffiths, Marieangela C. Wilson, Kathryn E. Mordue, Abi Gartner, Tatyana N. Andrienko, Annabel Calvert, Alison Condie, Angela McCahill, Joanne C. Mountford, Ashley M. Toye, David J. Anstee, Jan Frayne

**Affiliations:** 1 School of Biochemistry, University of Bristol, Bristol, BS8 1TD UK; 2 Department of Biochemistry, Faculty of Medicine Siriraj Hospital, Mahidol University, Bangkok, 10700 Thailand; 3 Bristol Institute for Transfusion Sciences, National Health Service Blood and Transplant (NHSBT), Bristol, BS34 7QH UK; 4 NIHR Blood and Transplant Research Unit, University of Bristol, Bristol, BS8 1TD UK; 5 Scottish National Blood Transfusion Service, Jack Copland Centre, Heriot Watt Research Park, Edinburgh, EH14 4AP UK

## Abstract

**Background:**

Pluripotent stem cells are attractive progenitor cells for the generation of erythroid cells in vitro as have expansive proliferative potential. However, although embryonic (ESC) and induced pluripotent (iPSC) stem cells can be induced to undergo erythroid differentiation, the majority of cells fail to enucleate and the molecular basis of this defect is unknown. One protein that has been associated with the initial phase of erythroid cell enucleation is the intermediate filament vimentin, with loss of vimentin potentially required for the process to proceed.

**Methods:**

In this study, we used our established erythroid culture system along with western blot, PCR and interegation of comparative proteomic data sets to analyse the temporal expression profile of vimentin in erythroid cells differentiated from adult peripheral blood stem cells, iPSC and ESC throughout erythropoiesis. Confocal microscopy was also used to examine the intracellular localisation of vimentin.

**Results:**

We show that expression of vimentin is turned off early during normal adult erythroid cell differentiation, with vimentin protein lost by the polychromatic erythroblast stage, just prior to enucleation. In contrast, in erythroid cells differentiated from iPSC and ESC, expression of vimentin persists, with high levels of both mRNA and protein even in orthochromatic erythroblasts. In the vimentin-positive iPSC orthochromatic erythroblasts, F-actin was localized around the cell periphery; however, in those rare cells captured undergoing enucleation, vimentin was absent and F-actin was re-localized to the enucleosome as found in normal adult orthrochromatic erythroblasts.

**Conclusion:**

As both embryonic and adult erythroid cells loose vimentin and enucleate, retention of vimentin by iPSC and ESC erythroid cells indicates an intrinsic defect. By analogy with avian erythrocytes which naturally retain vimentin and remain nucleated, retention in iPSC- and ESC-derived erythroid cells may impede enucleation. Our data also provide the first evidence that dysregulation of processes in these cells occurs from the early stages of differentiation, facilitating targeting of future studies.

**Electronic supplementary material:**

The online version of this article (10.1186/s13287-019-1231-z) contains supplementary material, which is available to authorized users.

## Introduction

The generation of red blood cells in vitro as an alternative clinical product is of interest to blood services globally. Peripheral blood, cord blood, induced pluripotent (iPSC) and embryonic stem cells (ESC) have been used as progenitors in erythroid culture systems, all differentiating along the erythroid pathway [1–5]. However, erythroid cells differentiated from adult peripheral blood and cord blood stem cells have a restricted expansion potential using current systems [6]. In contrast, pluripotent stem cells (ESC and iPSC) have the potential to provide an inexhaustible source of progenitors for the generation of large numbers of erythroid cells. In particular, exploration of iPSC as a progenitor source is attractive as they can be derived from easily accessible adult cells, and without the associated ethical issues of ESCs, opening up opportunities for autologous transfusion products. However, in comparison to the high proportion of enucleated reticulocytes achieved from adult and cord blood progenitors, up to 95% [2, 5], enucleation rates for erythroid cells differentiated from ESC and iPSC are low, ≤ 10% [1, 3, 4, 7, 8]. An increased yield of erythroid cells from iPSC and ESC has been achieved using a multi-step differentiation protocol to mimic and surpass the early stages of development; however, enucleation rates remained low [9]. Although a markedly higher enucleation rate for ESC line H1 has been reported in one paper [3], it could not be achieved for ESC line H9 in the same study, or for H1 in other studies [7]. The molecular basis of the enucleation defect therefore requires much further investigation to enable rectification before these cells can be considered as a reliable source for therapeutic applications.

Red blood cell enucleation is a continuous multi-step process (reviewed by Migliaccio and Keerthivasan et al. [10, 11]); the molecular details of which are still undefined, although recent advances have been made in elucidating the process [2, 10, 12, 13]. One protein that has been associated with the initial phase of enucleation is the intermediate filament vimentin, which forms part of the radial and juxtanuclear intermediate filament network. Vimentin plays an important role in supporting the intracellular organelles, especially the nucleus, with filaments extending from the nuclear periphery to the cell membrane, anchoring the nucleus in the cytoplasm of the cells [14].

Notwithstanding, in non-erythroid cells, vimentin’s role in orchestrating a wide range of cellular events is exemplified by its involvement in cell migration and adhesion [15, 16], interaction with signalling proteins [17] and in cytoskeleton cross-talk [18].

In murine erythroleukemia (MEL) cells, there is a marked and rapid loss of vimentin when the cells are chemically induced to differentiate [19]. Similarly, murine embryonic erythroid cells lose vimentin late in differentiation [20]; both these and human embryonic erythroid cells are now known to enucleate [21, 22] (reviewed by Palis 2014 [23]) and vimentin is absent in murine definitive erythrocytes [20]. There is little data in the literature addressing the expression of vimentin in human erythroblasts. One early study described vimentin unusually as non-filamentous in structure, and although absent in mature erythrocytes, its loss during erythroid maturation as random rather than related to a stage of differentiation [24]. In contrast, vimentin is retained in avian erythrocytes which are nucleated, anchoring the nucleus within the cell [25], suggesting a role for vimentin regulation in enucleation.

In this study, we show in adult erythropoiesis vimentin mRNA is lost early during the differentiation process, with a precipitous loss in protein levels between basophilic and polychromatic erythroblasts, prior to enucleation. In contrast in erythroid cells differentiated from the ESC line RC9 and iPSC line C19 expression of vimentin continues, with high levels of both mRNA and protein detected even in orthochromatic erythroblasts. In adult orthochromatic erythroblasts undergoing enucleation F-actin is re-located to the enucleosome structure. In contrast in the majority of vimentin-positive iPSC orthochromatic erythroblasts, F-actin remained localized around the cell periphery. However, in the very rare cells captured undergoing enucleation, vimentin was absent, and F-actin was localized to the same enucleosome structure. Although inconclusive because of the low enucleation rate and thus difficulty in capturing cells undergoing enucleation at any one time, it is tempting to speculate a link between vimentin retention and failure of actin re-localisation, the proteins being known to interact.

As both embryonic (primitive) and adult (definitive) erythroid cells enucleate and loose vimentin, retention of vimentin by erythroid cells differentiated from iPSC and ESC is an intrinsic defect. By analogy with avian erythrocytes which also retain vimentin and are nucleated, aberrant retention of vimentin in iPSC- and ESC-derived erythroid cells may impede their enucleation. Importantly, our data also provides the first indication that dysregulation of processes in these iPSC and ESC erythroid cells occurs from, at least, the early stages of erythroid differentiation.

## Materials and methods

### Cell isolation and culture

Haematopoietic differentiation of the ESC line RC9 [26] and iPSC line C19 [27] and isolation of CD34^+^ cells were performed as described previously [4, 9, 27]. Adult peripheral blood CD34^+^ cells were isolated as described previously [2].

Adult, C19 and RC9 CD34^+^ cells were cultured in a three-stage erythroid culture system [2]. In brief, the base medium consisted of Iscove’s modified Dulbecco’s medium (IMDM, Source BioScience, Nottingham, UK) containing 3% (*v*/*v*) AB Serum (Sigma-Aldrich, Poole, UK), 2% FCS (Hyclone; GE Healthcare SH30071.03), 10 μg ml^−1^ insulin (Sigma-Aldrich), 3 U ml^−1^ heparin (Sigma-Aldrich), 200 μg ml^−1^ transferrin (R&D Systems, Abingdon, UK) and 3 U ml^−1^ Epo (Roche, Welwyn Garden City, UK). The first stage was supplemented with 10 ng ml^−1^ stem cell factor (SCF, Medsafe, Sweden) and 1 ng ml^−1^ IL-3 (R&D Systems, Abingdon, UK), the second stage with 10 ng ml^−1^ SCF and the final stage with an additional 300 μg ml^−1^ transferrin. RC9 CD34^+^ cells were co-cultured with OP9 cells from days 0–7 in Stemline (Sigma) containing 1 μM hydrocortisone, 50 ng ml^−1^ SCF, 16.7 ng ml^−1^ Flt3L, 6.7 ng ml^−1^ BMP4, 6.7 ng ml^−1^ IL3, 6.7 ng ml^−1^ IL11, 3 U ml^−1^ EPO, 50 uM IBMX and 10% FCS. After day 7, cells were co-cultured with OP9 in Iscove (Biochrom) containing 1% BSA, 10 μg ml^−1^ insulin, 0.2 mg ml^−1^ transferrin, 0.1 mM β-mercaptoethanol, 1× lipid (peprotech), 1 μM hydrocortisone, 20 ng ml^−1^ SCF, 20 ng ml^−1^ IGF-1, 6.7 ng ml^−1^ IL3, 6.7 ng ml^−1^ IL11, 3 U ml^−1^ EPO and 10% FCS.

We have previously shown pluripotency proteins Oct-4, SOX-2 and KLF-4 are lost in erythroid cells differentiated from the C19 iPSC line [4], and expression of pluripotency markers SSEA4, SSEA3 and TRA 1-60 are lost from erythroid cells differentiated from the RC9 ESC line [9]. We have further compared the expression of Oct-4, SOX-2 and NANOG in erythroid cells differentiated from ESC lines RC9 and H1 with that of stage-matched adult erythroid cells, which shows high levels of expression in the undifferentiated stem cells, as expected, but by 10 days of erythroid differentiation levels are equivalent to that in the adult cells (Additional file 1: Figure S1).

Adult orthochromatic erythroblasts were isolated following incubation with tetramethylrhodamine methyl ester (TMRM). Cells at day 16 in culture were dual labelled with Hoechst 33342 (5 μg/ml) and TMRM (25 nM). Orthochromatic erythroblasts were detected by selected gating (primary gate Hoechst vs Forward scatter area; secondary—TMRM), and then isolated using a BDInflux Cell Sorter.

### Antibodies

Vimentin RV202 (ab8978; Abcam) for western blot, flow cytometry and confocal microscopy; α-Globin (D-16, Santa Cruz Biotechnology), Glycophorin A (CDVP, IBGRL, UK), Band 3 (BRIC170, IBGRL, UK), β-actin (Sigma), LC3B (Cell Signaling) and GABARAPL1 [28] for western blot; and Glycophorin A (BRIC256, IBGRL, UK) for confocal microscopy.

### Whole-cell lysate preparation

Cultured cells were re-suspended in solubilisation buffer (20 mM Tris HCl [pH 7.5], 150 mM NaCl, 10% glycerol, 1% Triton, 0.1% SDS, 1× complete protease inhibitor and 2 mM PMSF) and incubated for 1 h on ice before incubation with 25 U ml^−1^ Bensonase for 1 h at 4 °C. Samples were centrifuged at 17,000*g* for 5 min at 4 °C and supernatants collected. All chemicals were obtained from Sigma-Aldrich.

### Western blot

Membranes were blocked with 10% milk powder, incubated overnight at 4 °C with primary antibody and washed and incubated with secondary antibody (rabbit α-mouse immunoglobulin-HRP; DakoCytomation) for 1 h at room temperature. Bands were visualized using enhanced chemilunescence (G.E. Healthcare).

### PCR analysis

Primers (Sigma-Aldrich) used were forward: AAATGGCTCGTCACCTTCGT, reverse: TTGCGCTCCTGAAAAACTGC for vimentin and forward: ACCACAGTCCATGCCATCAC, reverse: TCCACCACCCTGTTGCTGTA for GAPDH with an annealing temperature of 58 °C and 30 cycles.

### Confocal microscopy

All procedures were as described previously [27].

### Comparative proteomics

Comparative proteomics was performed using Tandem Mass Tags (TMT) as described previously [5, 29] and analysed using an Orbitrap Fusion Tribrid mass spectrometer (Thermo Scientific). Only rank 1 peptides with high/medium confidence were used in analyses.

### Vimentin knockdown

Cells were transduced with pLKO.1 short hairpin (sh) RNA plasmids (sh19-23) against vimentin or with a scrambled (src) control shRNA plasmid (all designed by the Broad Institute and purchased from Open Biosystems, GE Dharmacon, Lafayette, CO, USA) for 24 h.

### Flow cytometry

Cells were fixed in 1% pararformaldehyde for 15 min before permeabilisation with 0.05% Triton X-100 for 5 mins. Cells were then blocked with 4% BSA before incubation with vimentin antibody followed by rat APC-conjugated anti-mouse IgG1 (Biolegend, London, UK) and analysed on a MACSQuant system (Miltenyi Biotech Ltd., Bisley, UK).

## Results

### Expression profile of vimentin during adult erythropoiesis

**Fig. 1:**
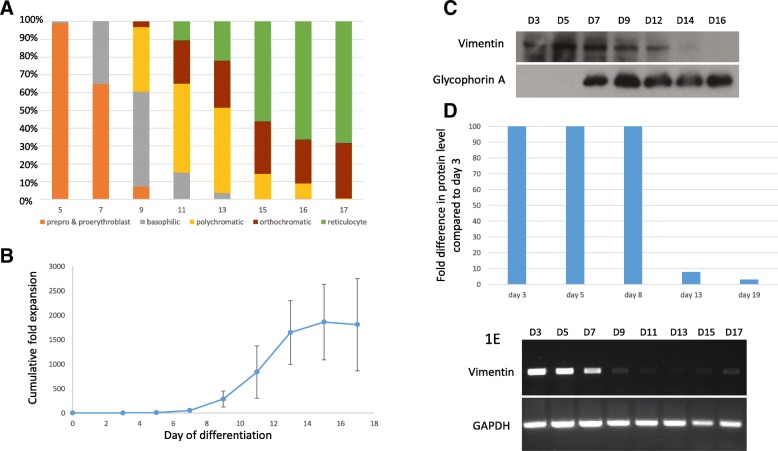
Expression profile of vimentin during adult erythropoiesis Adult peripheral blood CD34^+^ cells were incubated for up to 21 days in a three-stage erythroid culture system. **a** Cells were stained with May-Grünwald-Giemsa reagent at time points throughout the culture (see also Additional file 1: Figure S1A) and the proportion of cells (*Y*-axis) at different stages of differentiation counted (data is representative of three cultures). **b** Cell numbers at time points through erythropoiesis were counted and the cumulative fold expansion calculated *n* = 3 ± S.D. **c** Western blot of adult erythroid cells at different days in culture probed with antibody to vimentin. An antibody to Glycophorin A was used as a control. **d** The abundance of vimentin peptides at different time points in culture was compared by labelling with TMTs and analysis by nano LC-MS/MS. Vimentin was quantified using 20 peptides with 47 PSM. **e** The abundance of vimentin transcripts at time points throughout erythroid culture was analysed by PCR. Abundance of GAPDH transcripts was used as a control

To examine the expression profile of vimentin during adult erythropoiesis, CD34^+^ cells isolated from peripheral blood were incubated in our three-stage culture system which has previously been shown to achieve efficient erythroid differentiation [2]. Morphological analysis from day 5 (Fig. 1a, Additional file 1: Figure S2A) confirmed erythroid differentiation. By day 21 of culture, ~ 90% enucleation was routinely achieved. Maximum proliferation occurred at the pro-erythroblast stage of differentiation, but continued until approximately day 15 (Fig. 1b), in line with a final mitosis at the polychromatic stage as determined previously [30]. We first examined the relationship between vimentin expression and erythroid differentiation by western blot. We found that vimentin was present in cells at the early stages of erythroid differentiation but then lost, coinciding temporally with differentiation of basophilic to polychromatic erythroblasts (Fig. 1c). We also examined the abundance of vimentin by comparative proteomics. Tryptic peptides generated from cells at days 3, 5, 8, 13 and 19 in culture were labelled with different Tandem Mass Tags (TMT) and analysed by nano LC-MS/MS, using methodology described previously [5]. In line with the western blot, a sharp drop (13-fold) in the level of vimentin occurred between cells at day 8 in culture when the predominant cell type is basophilic, and day 13 when the predominant cell type is polychromatic (Fig. 1d). Negative controls, i.e. proteins that did not change in level between day 8 to 13, include AHSP, ankyrin 1, aquaporin 1, band 4.2, carbonic anhydrase 1, catalase, spectrin α and β and tubulin β, as well as Glycophorin A in line with the western blot data in Fig. 1c. We also interrogated our proteomic data set for cells following enucleation (data available in Wilson et al. [5]), detecting no vimentin peptides in reticulocytes or endogenous mature RBCs. A similar expression profile and magnitude decrease (18-fold) in vimentin abundance on differentiation of early basophilic to polychromatic erythroblasts was found on interrogating the proteome dataset generated by Gautier et al. [31], who compared the level of proteins in adult erythroid cells isolated at distinct stages of differentiation in vitro using label-free quantification. Vimentin protein is thus clearly lost when cells are undergoing the final stages of terminal differentiation, just prior to enucleation.

As vimentin is regulated at the transcriptional level [19, 32], we also analysed the expression profile of vimentin mRNA during adult erythropoiesis. Transcripts were detected in early erythroid cells, but expression then ceased (Fig. 1e), temporally coinciding with differentiation of pro-erythroblasts to basophilic erythroblasts.

### Expression of vimentin in erythroid cells differentiated from ESC and iPSC

Avian erythroid cells are nucleated and retain vimentin [25]. We therefore questioned whether erythroid cells differentiated from ESC and iPSC also retain vimentin. We have previously shown that CD34^+^ cells differentiated from ESC line RC9 and iPSC line C19, used in the present study, undergo erythroid differentiation and express key erythroid markers, with the majority of cells failing to enucleate [4, 9].

**Fig. 2:**
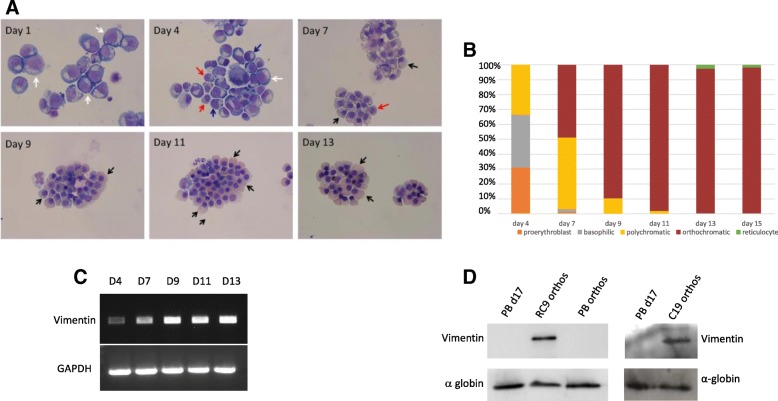
Expression of vimentin in erythroid cells differentiated from the ESC line RC9 RC9 CD34^+^ cells were incubated for up to 19 days in a three-stage erythroid culture system. **a** Cells were stained with May-Grünwald-Giemsa reagent at time points throughout the culture. White arrows, pro-erythroblasts; blue arrows, basophillic erythroblasts; red arrows, polychromatic erythroblasts; black arrows, orthochromatic erythroblasts. (**b**) The proportion of cells (*Y*-axis) at different stages of differentiation counted (data is representative of three cultures). **c** The abundance of vimentin transcripts at time points throughout erythroid culture was analysed by PCR. Abundance of GAPDH transcripts was used as a control. **d** Cells at day 17 in adult culture (orthochromatic erythroblasts and reticulocytes), isolated adult orthochromatic erythroblasts, RC9 and C19 orthochromatic erythroblasts were probed with an antibody to vimentin. Blots were stripped, and an α-globin antibody used as a control for protein loading

Following the haematopoietic differentiation of RC9 cells in the present study, CD34^+^ cells were isolated and transferred to our erythroid culture system. The cells differentiated along the erythroid pathway (Fig. 2a, b; expression of GPA on day 13 in comparison with adult erythroid cells is shown in Additional file 1: Figure S2B) to orthochromatic erythroblasts, but the majority failed to enucleate, as expected. Prolonged time in culture did not result in any further enucleation; the cells instead died. The expression of vimentin transcripts was analysed by PCR during erythroid differentiation, with expression retained throughout even in orthochromatic erythroblasts (Fig. 2c). We confirmed the presence of vimentin protein in the RC9-derived orthochromatic erythroblasts by western blot (Fig. 2d), comparing with cells at day 17 in adult culture and with isolated adult orthochromatic erythroblasts (Additional file 1: Figure S2C); the latter included for consistency as at day 17 there is a mixed population of orthochromatics and reticulocytes present in adult cultures. No vimentin was detected in the adult samples. We also detected vimentin in orthochromatic erythroblasts differentiated from the iPSC line C19 (Fig. 2d) using the same culture system (Additional file 1: Figure S3 shows erythroid cell morphology of C19 cells during differentiation). In addition, comparative proteomics of C19 and adult pro-erythroblasts and of C19 and adult isolated orthochromatic erythroblasts show vimentin at an equivalent level in the pro-erythroblasts but approximately 20-fold higher in the iPSC compared to adult orthochromatic erythroblasts, supporting the data from western blot; vimentin was quantified from 30 and 34 unique peptides with 75 and 107 PSM respectively for these analyses. Hence, vimentin is retained in both the ESC-derived and iPSC-derived erythroid cells throughout erythropoiesis, with the transcript data indicting dysregulation from the early stage of terminal differentiation. Of note, we have shown previously that both culture systems used in this study result in the production of definitive, not primitive, erythroid cells from both iPSC and ESC [4, 9]. Erythroid cells differentiated from the RC9 line differentiate a little more rapidly than from the C19 line, as shown by the morphological analysis (Fig. 2a, b, Additional file 1: Figure S3A and B). However, erythroid cells from both lines achieve efficient differentiation to orthochromatic erythroblasts (Fig. 2a, b, Additional file 1: Figure S3A and B) with the level of GPA, Band 3 (key erythroid differentiation markers) and α-globin increasing during differentiation as expected (Additional file 1: Figure S4).

### Localisation of vimentin in erythroid cells

**Fig. 3:**
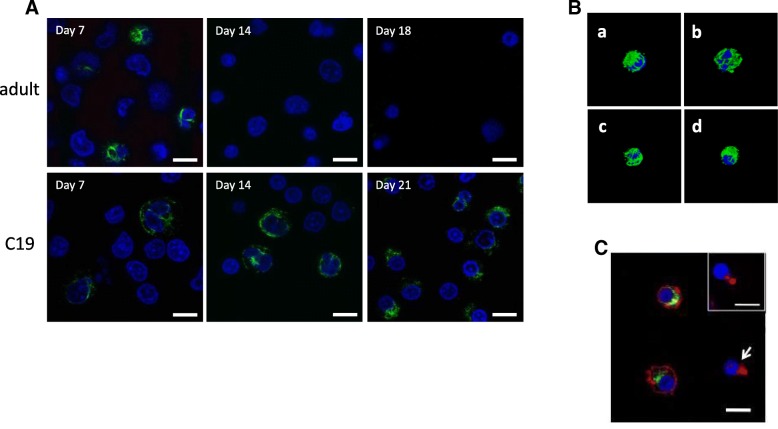
Localisation of vimentin in adult and C19 erythroid cells. The expression and conformation of vimentin in adult and C19 erythroblasts was analysed by confocal microscopy on days 7, 14 and 21 of culture. **a** Cells were probed with a vimentin antibody, followed by Alexa Fluor 488 (green). Nuclear DNA was stained with blue-fluorescent DAPI. Images are representative of the overall cultures. **b** 3D reconstruction from the images of adult erythroblasts on day 7 (a) and C19 erythroblasts on day 7 (b), 14 (c) and 21 (d). Images were reconstructed using velocity 6.1.1 software. **c** Orthochromatic erythrobasts from C19 cultures were incubated with vimentin antibody, followed by Alexa Fluor 488 (green) and Alexa Fluor 635 phalloidin (red). Arrow indicates an enucleating erythroblast with enucleosome structure formation. Inset shows an adult enucleating erythroblast with enucleosome. Scale bars 10 μm

We also looked at the conformation of vimentin in adult and C19 erythroblasts by confocal imaging on days 7, 14 and 21 of culture. Vimentin was detected in adult erythroblasts only on day 7, but in C19 erythroblasts at all time points. In all cells, detected vimentin appeared filamentous in structure, surrounding the nucleus (Fig. 3A). This is more clearly seen in the 3D reconstruction images shown in Fig. 3B for an adult erythroblast on day 7 (a), and C19 erythroblasts on days 7 (b), 14 (c) and 21 (d). In both the adult and C19 erythroblast populations, only 20–30% vimentin-positive cells were routinely detected. Heterogeneity in vimentin detection in populations of erythroid and MEL cells has been observed previously [24] and may be due to antigen masking or modification. Expression and conformation of vimentin in erythroid cells differentiated from a second iPSC line; OPM2 [4, 27] showed the same profile as the C19 erythroblasts at the same time points in culture by confocal analysis (data not shown).

Previous studies have shown the formation of an F-actin structure [2, 29], more recently termed an enucleosome [13], at the rear of the nucleus in enucleating adult orthochromatic erythroblasts, which is believed to generate the force required for nuclear extrusion. This structure was observed in enucleating adult orthochromatic erythroblasts in our present study (example is shown in Fig. 3C insert). In C19 orthochromatic erythroblasts at day 21 in culture co-labelled for vimentin and F-actin, in vimentin-positive cells, F-actin was localized around the cell periphery (Fig. 3C). However, in the rare cells captured undergoing enucleation, vimentin was absent, and F-actin was localized to a similar enucleosome structure (Fig. 3C, arrow). Although inconclusive because of the low enucleation rate and thus extremely low number of cells undergoing enucleation at any one time, it is tempting to speculate a link between vimentin retention and failure of actin re-localisation, as the two proteins are known to interact [33, 34]. Thus, a small sub population of C19 erythroid cells may be correctly programmed and are thus able to enucleate normally.

### Knockdown of vimentin in C19 erythroid cells

Finally, we knocked down vimentin mRNA in C19 erythroid cells, selecting the time in culture when the majority of cells were basophilic, to correlate with the stage of differentiation vimentin transcripts are naturally lost in adult cells (Fig. 1e). The efficiency of five vimentin shRNAs was first verified in K562 cells (Additional file 1: Figure S5A and B) with shRNA 21 reducing vimentin protein by the greatest amount (~ 80%). C19 erythroid cells were transduced with this hairpin and knock down verified by fluorescent microscopy. Unfortunately, vimentin knockdown stopped the cells dividing resulting in cell death (Additional file 1: Figure S5C); thus, we were unable to determine an effect on enucleation. As vimentin loss occurs naturally in adult erythroblasts and is clearly not detrimental, presumably complexes and processes involving vimentin [17, 18, 33, 34] undergo prior or parallel reorganization, no longer requiring vimentin. In contrast in C19 erythroid cells, vimentin-dependent complexes may persist alongside vimentin, resulting in their disruption when vimentin is knocked down and thus the observed cell death. Simply knocking vimentin down or out in these cells is therefore not a solution to correct the enucleation defect; instead, the underlying dysregulation in these cells needs to be determined.

### Correlation of miR-30a expression with enucleation

To investigate the molecular basis of the enucleation defect of ESC-derived erythroid cells, Rouzbeh et al. [35] analysed gene and miRNA expression profiles, reporting miR-30a as a key regulator of erythroblast enucleation with aberrant overexpression responsible for the defect. They showed that erythroid cells differentiated from ESC line H1 day 20 embryoid bodies (EBs) achieved a high enucleation rate of ~ 55%, but when differentiated from day 9 EBs the rate dropped to ~ 1%. In cells differentiated from day 9 EBs, miR-30a was aberrantly elevated in late-stage erythroid cells. Subsequent knockdown of miR-30a in these cells increased the enucleation rate to ~ 51%. However, using a second ESC line, H9, enucleation rates of erythroid cells differentiated from day 20 EBs were only ~ 11%, in line with that commonly achieved for many ESC and iPSC lines. Notwithstanding, enucleation rates of erythroid cells differentiated from H9 day 9 EBs of < 1% were increased to ~ 10% on knockdown of miR-30a. Although not as striking, aberrant expression of this miRNA may contribute to the defective enucleation process in these cells.

**Fig. 4:**
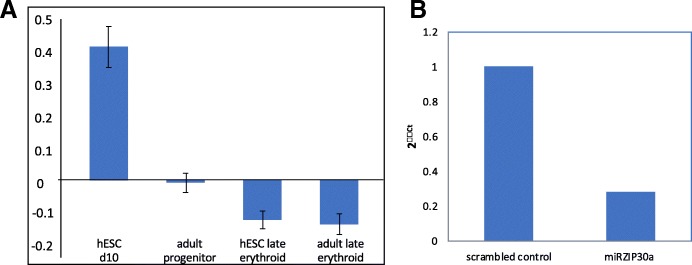
Expression and knockdown of miR-30a. **a** miR-30a microarray expression data from hESC and adult samples matched for developmental stage. Total microRNA was processed and analysed by Sistemic Ltd., using the Agilent miRNA platform (using version 3 of Agilent’s Human microRNA microarray slides; miRBase version 12.0), *n* = 4 ± SE. **b** miR30a expression as assessed by real-time quantitative polymerase chain reaction in cells transduced with miRZIP-30a and a scrambled vector at 17 days post-transduction. Relative fold change in expression (normalized to RNU48) was calculated by the ΔΔCT method, and values are expressed as 2ΔΔCT. The plot is representative of two repeats

Interestingly, in other cell types, miR-30a has been shown to downregulate the expression of vimentin [36–38]. We therefore also investigated the expression of miR-30a in erythroid cells differentiated from H1 early EBs but did not find aberrantly elevated levels of miR-30a, the expression profile during erythropoiesis declining in line with that in adult erythroid cells (Fig. 4a) and in erythroid cells from day 20 EB bodies in the Rouzbeh et al. study [35]. The level of miR-30a was however higher in the EBs than that in stage-matched adult progenitors [8] (Fig. 4a). We therefore used the same methodology as Rouzbeh et al. [35] to knock down miR-30a in erythroid cells differentiated from day 9 EBs and achieved reduced levels of miR-30a that were retained throughout erythroid culture (Fig. 4b), but enucleation remained negligible. Confirmation of the effect of miR-30a knockdown was achieved by verifying increased levels of miR-30a targets GABARAPL1 and LC3B by western blot (Additional file 1: Figure S6).

During adult erythropoiesis, vimentin transcript (Fig. 1e) and miR-30a levels (Fig. 4a) show a similar expression profile, with levels highest in early erythroid cells and rapidly declining as cells differentiate further. The miR-30a profile is also similar in cord blood erythroid cells during erythropoiesis [35]. MiR-30a may therefore play a role in degradation of vimentin transcript in these cells. However, any such association is clearly lost in erythroid cells differentiated from ESCs, or the regulation of vimentin by miR-30a could be context dependent.

The discrepancy between our data and that of Rouzbeh et al. may be due to different clones of H1 having different expression profiles and thus behaviour in culture; however, our data demonstrate that modulation of miR30a alone is not sufficient to promote enculeation in ESC erythroid cells. Comparison between the clone with high erythroid enucleation potential and those with low enucleation rates could potentially be informative.

## Discussion

In this study, we show that during normal adult human erythropoiesis expression of vimentin is switched off early during differentiation with the level of vimentin protein declining strikingly at the late stage of terminal differentiation, just prior to enucleation. In contrast, vimentin expression is maintained and vimentin protein retained in erythroid cells differentiated from ESCs and iPSCs. This clearly does not prevent these cells differentiating to orthochromatic erythroblasts but may impede the enucleation process resulting in or contributing to the poor enucleation rates achieved for these cells.

Vimentin is also retained in avian erythrocytes, which are nucleated, attributed to differences in cis-regulatory elements between the mammalian and avian vimentin gene [39]. Chicken vimentin mRNA levels increased significantly on the differentiation of MEL cells transfected with the entire chicken vimentin gene, whereas in cells transfected with the hamster vimentin gene the corresponding mRNA levels declined in line with the endogenous murine vimentin mRNA. Conversely, in a separate study, chicken vimentin was not detected in mature erythrocytes from transgenic mice expressing the chicken vimentin gene [40]. The regulatory mechanism for retention of vimentin in avian erythroid cells is therefore unresolved but does raise potentially interesting parallels with vimentin and nuclear retention in iPSC- and ESC-derived erythroid cells.

Notwithstanding, retention of vimentin, and other anomalies of iPSC- and ESC-derived erythroid cells, may not be due to inherent defects of the cells but to insufficiencies in the culture systems used. Our erythroid culture system supports efficient differentiation of adult and cord blood stem cells, with up to 95% and 85% enucleation respectively. Nevertheless, iPSC- and ESC-derived CD34^+^ cells may require other factors to induce correct terminal erythroid differentiation and thus enucleation. It is also possible that conditions during haematopoietic differentiation do not induce correct programming of the resultant CD34^+^ cell, with greater phenotypic analysis of these cells required. Either way, whether inherent or environmental our data indicate that defective programming of iPSC and ESC occurs early in erythroid differentiation and may occur even earlier during haematopoietic differentiation.

## Conclusion

In conclusion, we show vimentin gene expression ceases around the basophilic stage of differentiation in adult erythropoiesis. Persistent expression of the vimentin gene in iPSC and ESC erythroid cells from this stage indicates dysregulation of transcription from at least this stage of differentiation. In addition, selective nuclear reorganization in erythroblasts, whereby selected genes such as α- and β-globin and protein 4.1R continue to be transcribed right up until nuclear extrusion despite major transcriptional shut down [41], may be dysregulated in iPSC and ESC erythroid cells enabling genes such as vimentin to continue being transcribed to this late stage.

Clearly, further investigation into the regulation of gene and protein expression in iPSC- and ESC-derived erythroid cells is required to understand and rectify their enucleation defect. However, our data provide the first evidence that dysregulation occurs at the early stages of differentiation, helping direct future studies.

## Additional file

Additional file 1: Supplementary figures and legends. Vimentin expression is retained in erythroid cells differentiated from human iPSC and ESC and indicates dysregulation in these cells early in differentiation. (PPTX 28388 kb)

## Abbreviations

EB: Embryoid body
ESC: Ebryonic stem cell
iPSC: Induced pluripotent stem cell
miR: Micro RNA
mRNA: Messenger RNA
PCR: Polymerase chain reaction
TMT: Tandem Mass Tags
