# Supplementary material for: Vimentin expression is retained in erythroid cells differentiated from human iPSC and ESC and indicates dysregulation in these cells early in differentiation
Source: Stem Cell Res Ther. 2019 Apr 29;10:130. doi: 10.1186/s13287-019-1231-z (PMC6489253; doi:10.1186/s13287-019-1231-z)
Supplement: Supplementary file 1 — Supplementary figures and legends. Vimentin expression is retained in erythroid cells differentiated from human iPSC and ESC and indicates dysregulation in these cells early in differentiation. (PPTX 28388 kb) [file 13287_2019_1231_MOESM1_ESM.pptx]

## Slide 1
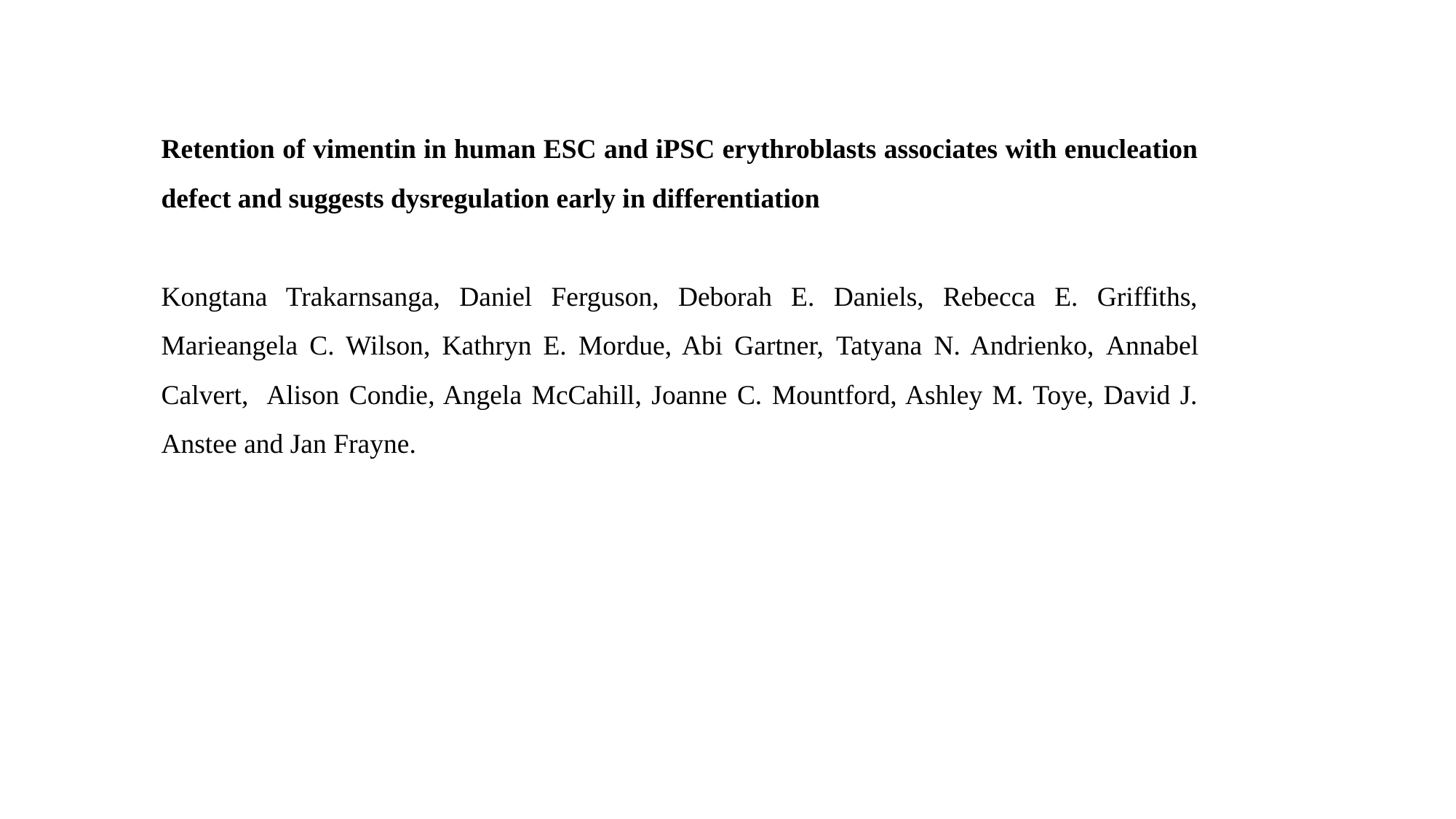

Retention of vimentin in human ESC and iPSC erythroblasts associates with enucleation defect and suggests dysregulation early in differentiation
Kongtana Trakarnsanga, Daniel Ferguson, Deborah E. Daniels, Rebecca E. Griffiths, Marieangela C. Wilson, Kathryn E. Mordue, Abi Gartner, Tatyana N. Andrienko, Annabel Calvert, Alison Condie, Angela McCahill, Joanne C. Mountford, Ashley M. Toye, David J. Anstee and Jan Frayne.

## Slide 2
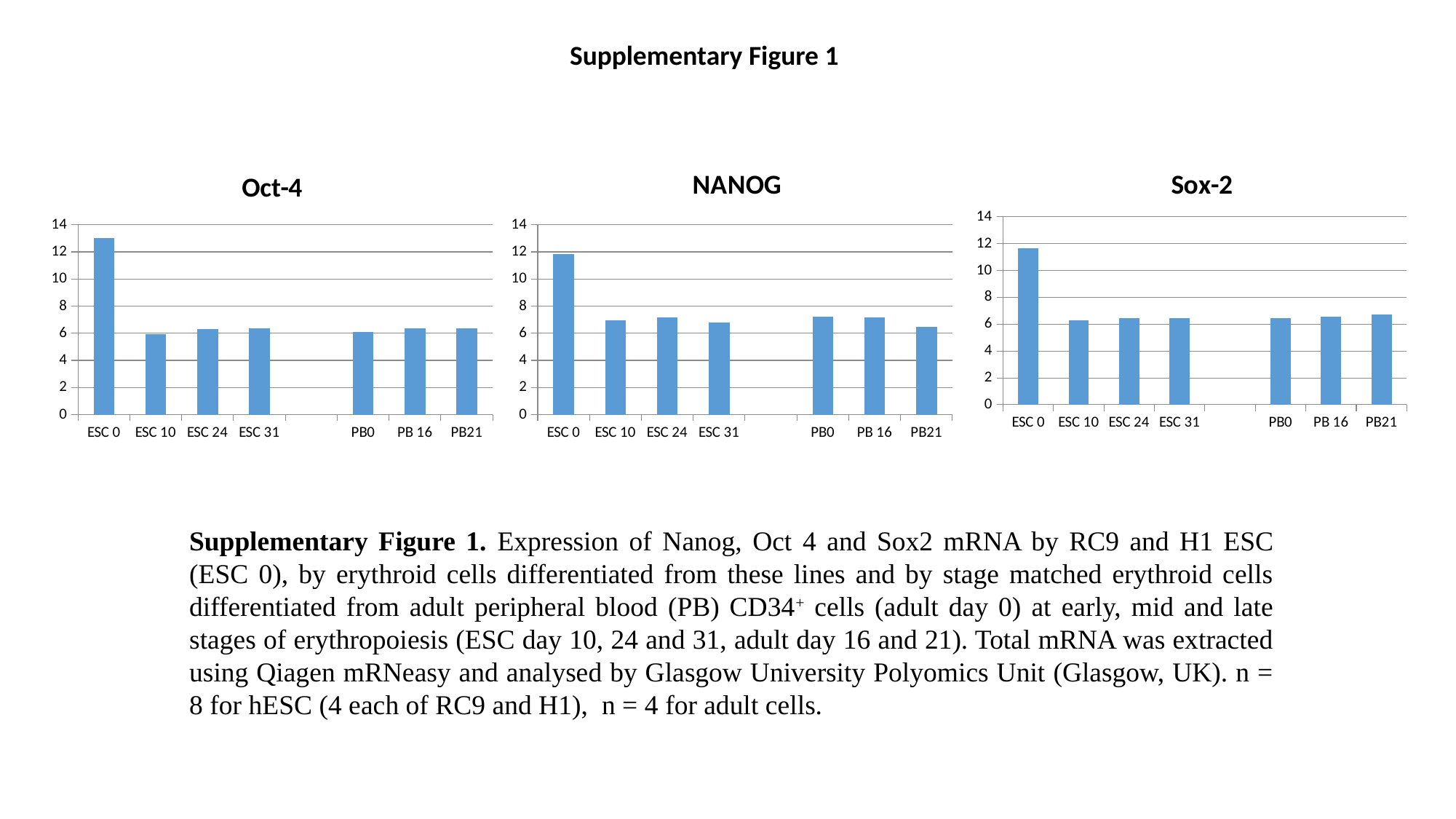

Supplementary Figure 1
### Chart: Sox-2
| Category | SOX2 |
|---|---|
| ESC 0 | 11.6705296433535 |
| ESC 10 | 6.26056467430293 |
| ESC 24 | 6.46984964078716 |
| ESC 31 | 6.45097509594884 |
| | None |
| PB0 | 6.43627440139414 |
| PB 16 | 6.57813514174937 |
| PB21 | 6.71094090170437 |
### Chart: Oct-4
| Category | POU5F1 |
|---|---|
| ESC 0 | 13.0062663155015 |
| ESC 10 | 5.93372368827741 |
| ESC 24 | 6.28223090387052 |
| ESC 31 | 6.36923789228922 |
| | None |
| PB0 | 6.10910067900267 |
| PB 16 | 6.34039298475632 |
| PB21 | 6.36890634596958 |
### Chart:
| Category | NANOG |
|---|---|
| ESC 0 | 11.8398812092795 |
| ESC 10 | 6.96578934250486 |
| ESC 24 | 7.14559513687523 |
| ESC 31 | 6.80309182579998 |
| | None |
| PB0 | 7.23519006499028 |
| PB 16 | 7.18078180194132 |
| PB21 | 6.48728870147877 |Supplementary Figure 1. Expression of Nanog, Oct 4 and Sox2 mRNA by RC9 and H1 ESC (ESC 0), by erythroid cells differentiated from these lines and by stage matched erythroid cells differentiated from adult peripheral blood (PB) CD34+ cells (adult day 0) at early, mid and late stages of erythropoiesis (ESC day 10, 24 and 31, adult day 16 and 21). Total mRNA was extracted using Qiagen mRNeasy and analysed by Glasgow University Polyomics Unit (Glasgow, UK). n = 8 for hESC (4 each of RC9 and H1), n = 4 for adult cells.

## Slide 3
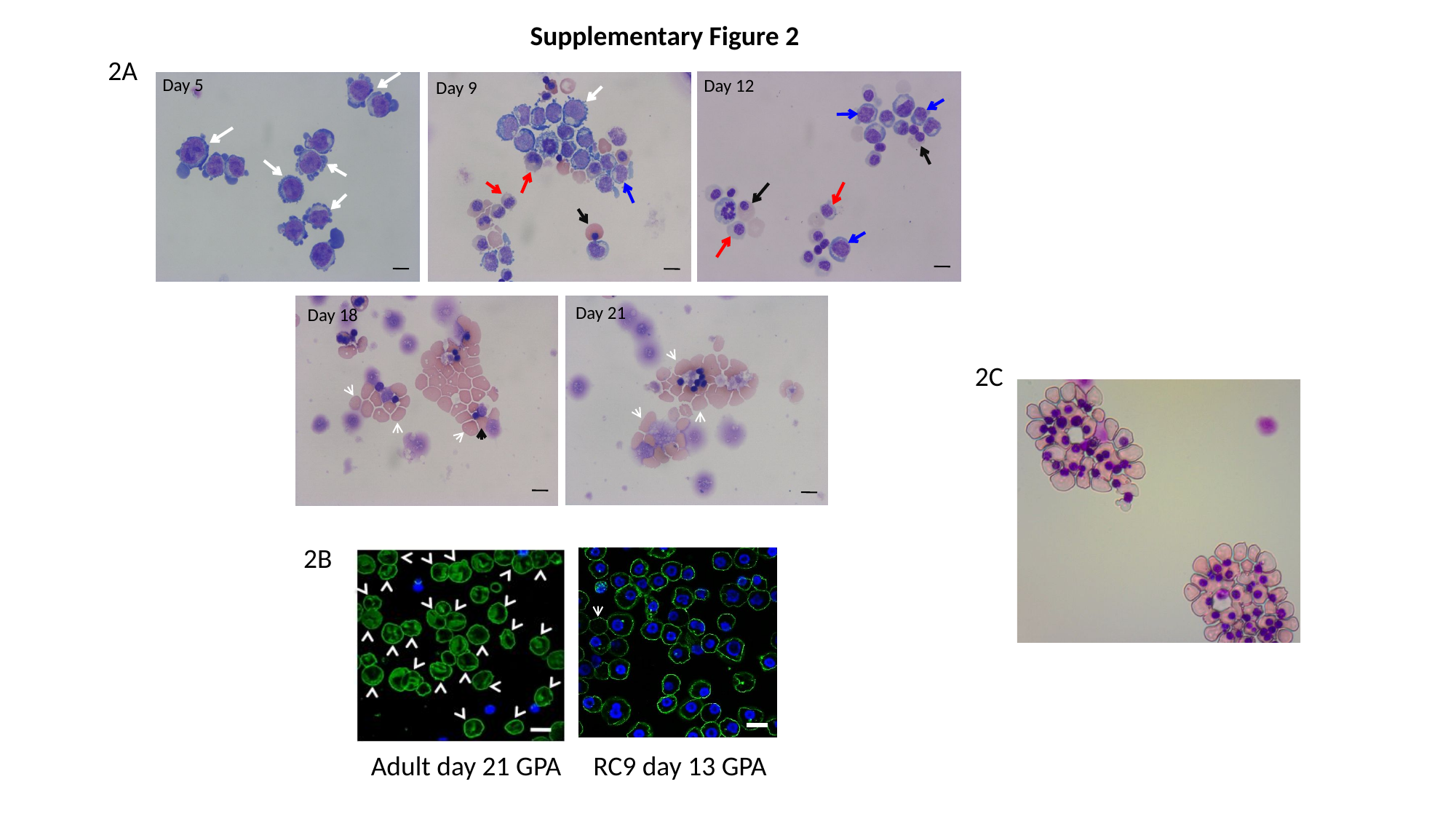

Supplementary Figure 2
2A
Day 5
Day 12
Day 9
Day 21
Day 18
2C
2B
RC9 day 13 GPA
Adult day 21 GPA

## Slide 4
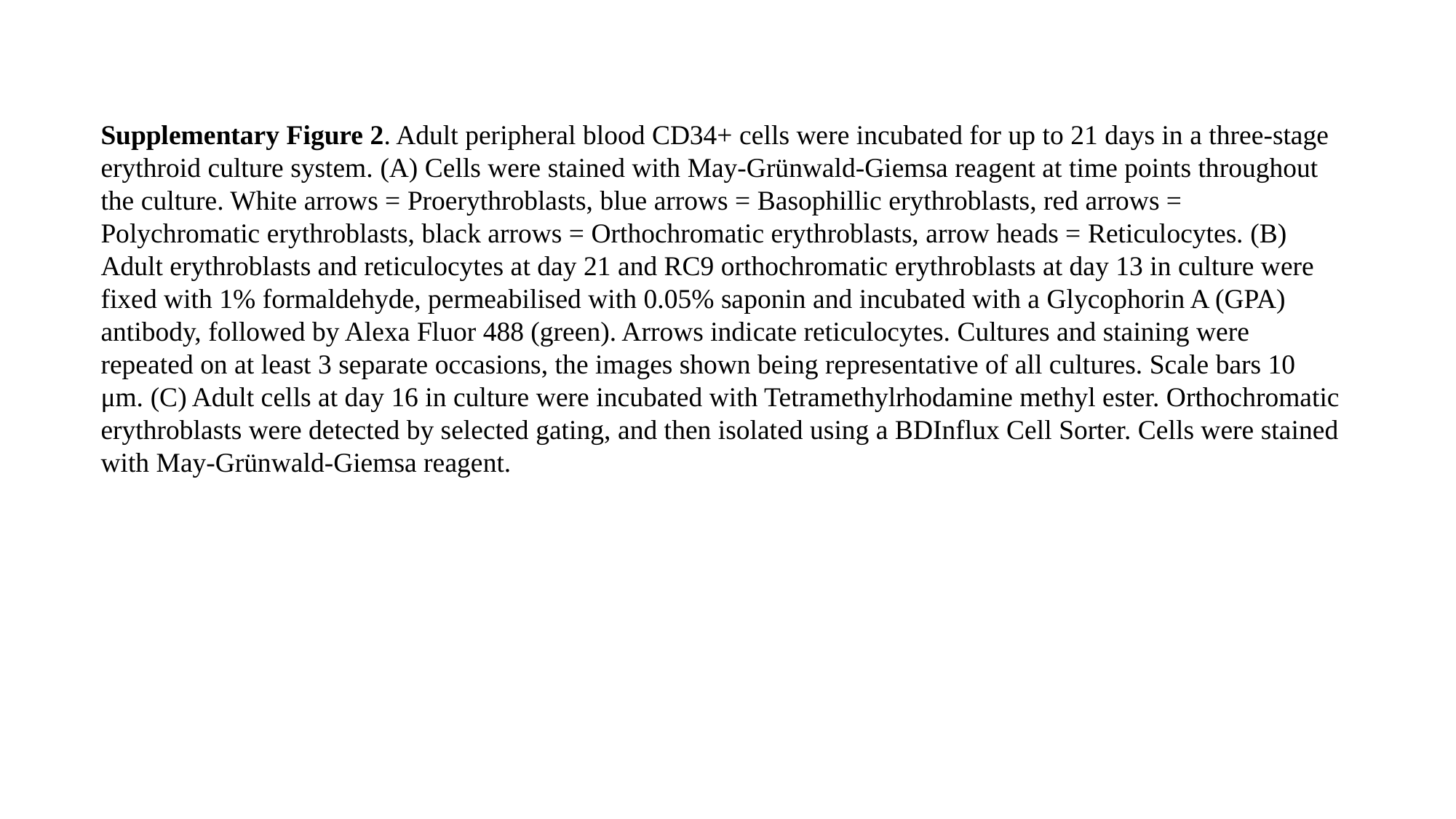

Supplementary Figure 2. Adult peripheral blood CD34+ cells were incubated for up to 21 days in a three-stage
erythroid culture system. (A) Cells were stained with May-Grünwald-Giemsa reagent at time points throughout
the culture. White arrows = Proerythroblasts, blue arrows = Basophillic erythroblasts, red arrows =
Polychromatic erythroblasts, black arrows = Orthochromatic erythroblasts, arrow heads = Reticulocytes. (B)
Adult erythroblasts and reticulocytes at day 21 and RC9 orthochromatic erythroblasts at day 13 in culture were
fixed with 1% formaldehyde, permeabilised with 0.05% saponin and incubated with a Glycophorin A (GPA)
antibody, followed by Alexa Fluor 488 (green). Arrows indicate reticulocytes. Cultures and staining were
repeated on at least 3 separate occasions, the images shown being representative of all cultures. Scale bars 10
μm. (C) Adult cells at day 16 in culture were incubated with Tetramethylrhodamine methyl ester. Orthochromatic
erythroblasts were detected by selected gating, and then isolated using a BDInflux Cell Sorter. Cells were stained
with May-Grünwald-Giemsa reagent.

## Slide 5
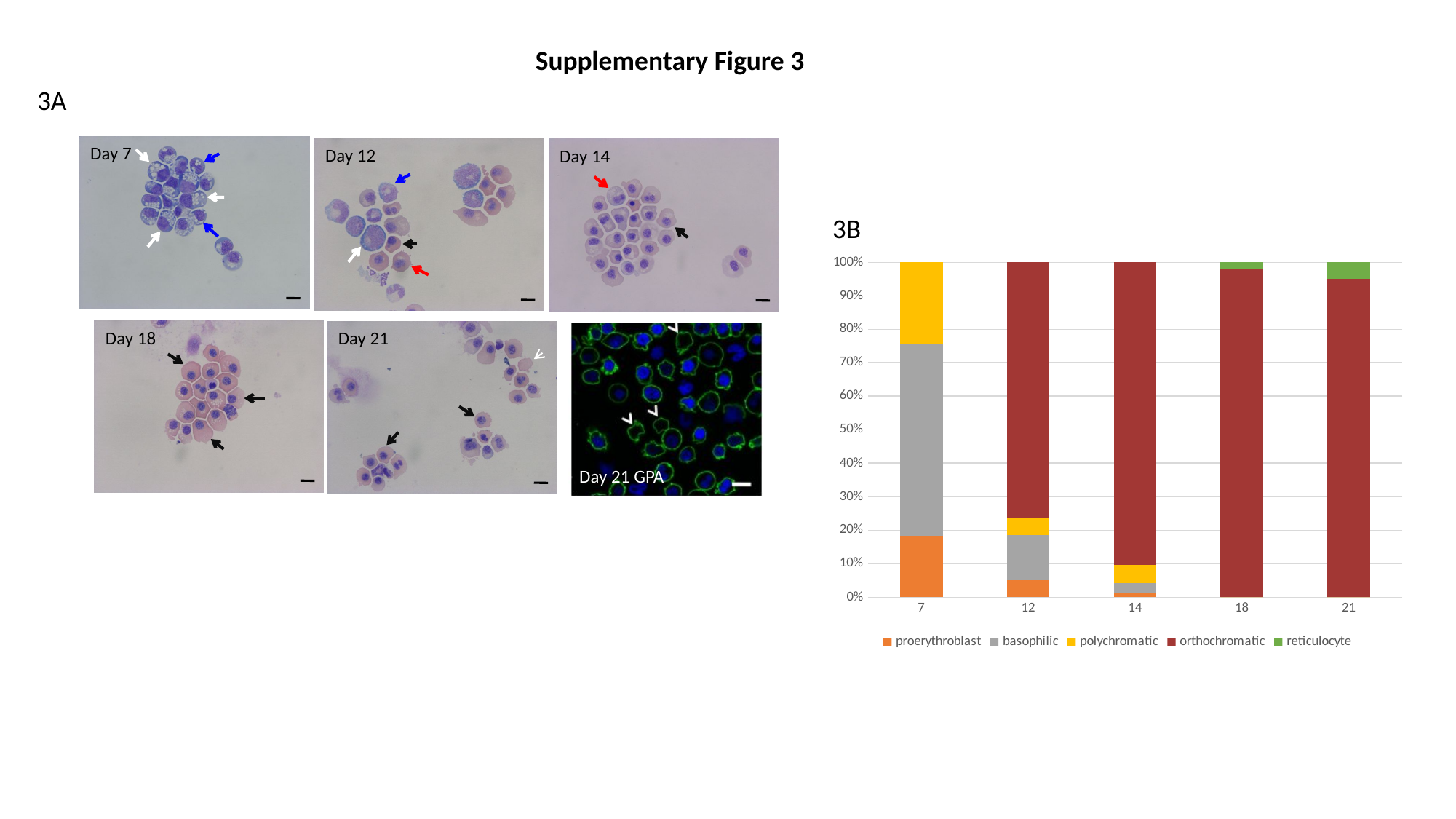

Supplementary Figure 3
3A
Day 7
Day 12
Day 14
Day 18
Day 21
3B
### Chart
| Category | proerythroblast | basophilic | polychromatic | orthochromatic | reticulocyte |
|---|---|---|---|---|---|
| 7 | 18.3 | 57.5 | 24.2 | 0.0 | 0.0 |
| 12 | 5.1 | 13.5 | 5.1 | 76.3 | 0.0 |
| 14 | 1.4 | 2.7 | 5.6 | 90.3 | 0.0 |
| 18 | 0.0 | 0.0 | 0.0 | 98.0 | 2.0 |
| 21 | 0.0 | 0.0 | 0.0 | 95.0 | 5.0 |
Day 21 GPA

## Slide 6
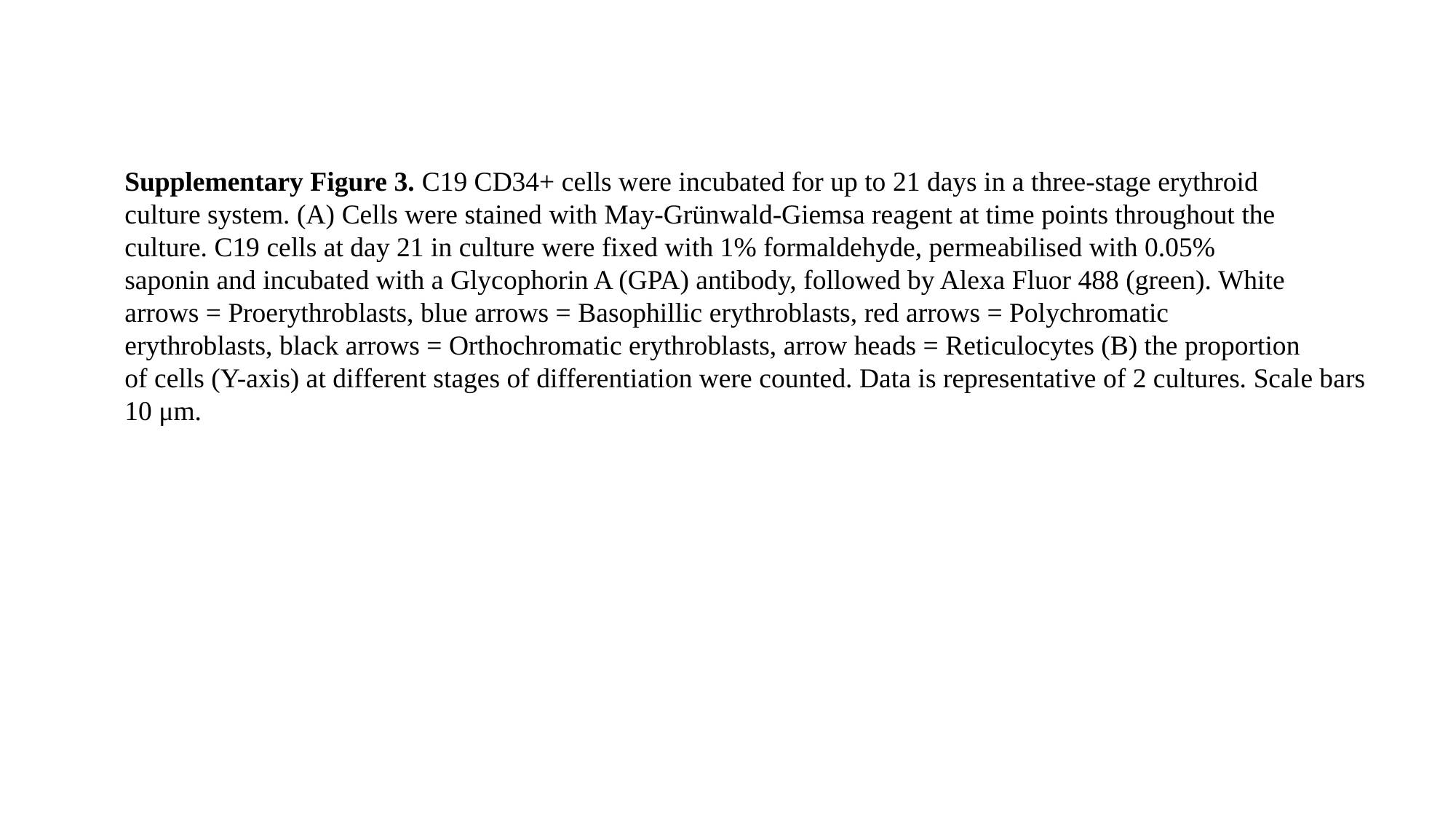

Supplementary Figure 3. C19 CD34+ cells were incubated for up to 21 days in a three-stage erythroid
culture system. (A) Cells were stained with May-Grünwald-Giemsa reagent at time points throughout the
culture. C19 cells at day 21 in culture were fixed with 1% formaldehyde, permeabilised with 0.05%
saponin and incubated with a Glycophorin A (GPA) antibody, followed by Alexa Fluor 488 (green). White
arrows = Proerythroblasts, blue arrows = Basophillic erythroblasts, red arrows = Polychromatic
erythroblasts, black arrows = Orthochromatic erythroblasts, arrow heads = Reticulocytes (B) the proportion
of cells (Y-axis) at different stages of differentiation were counted. Data is representative of 2 cultures. Scale bars
10 μm.

## Slide 7
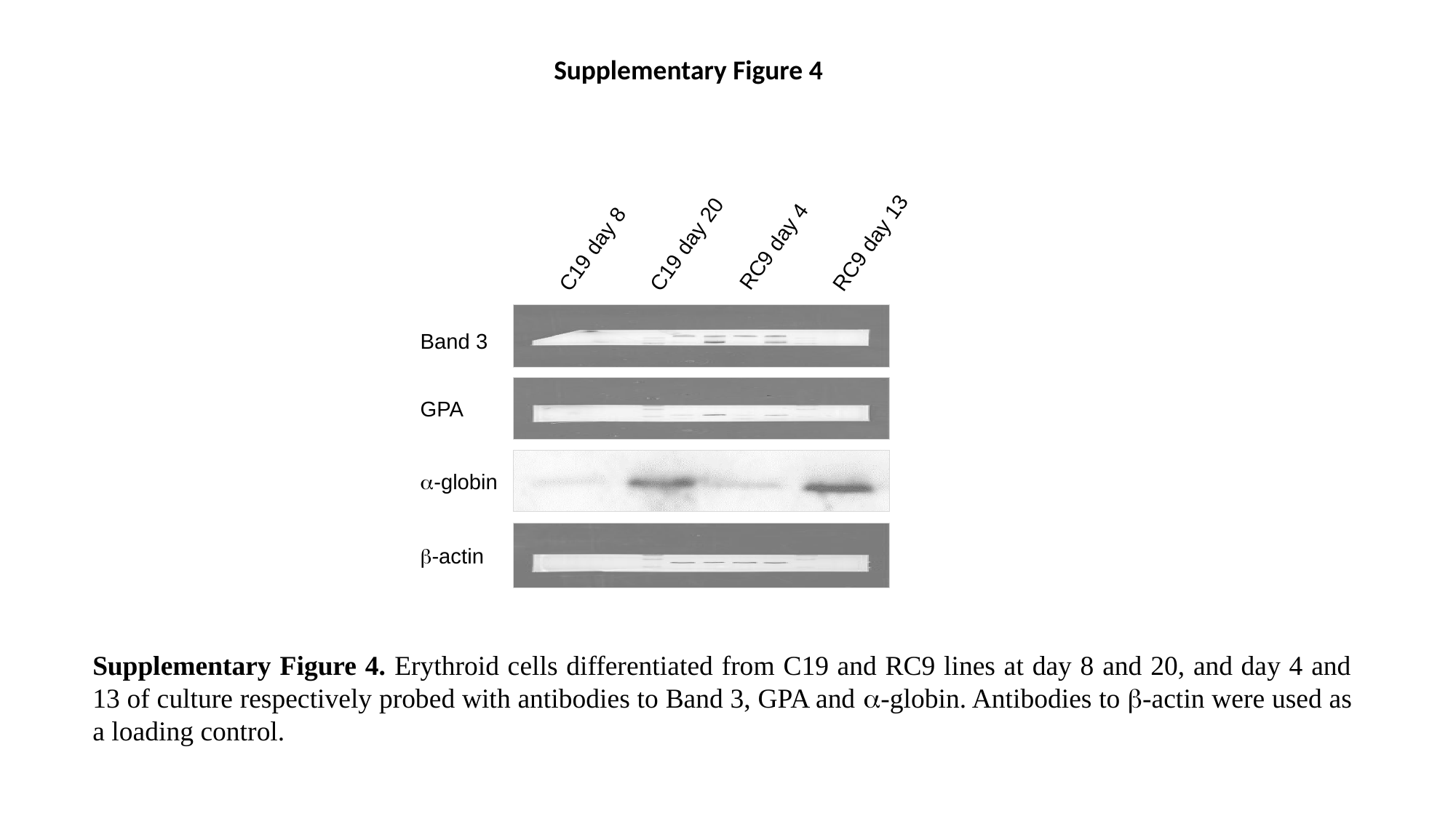

Supplementary Figure 4
RC9 day 4
RC9 day 13
C19 day 20
C19 day 8
Band 3
GPA
α-globin
β-actin
Supplementary Figure 4. Erythroid cells differentiated from C19 and RC9 lines at day 8 and 20, and day 4 and 13 of culture respectively probed with antibodies to Band 3, GPA and α-globin. Antibodies to β-actin were used as a loading control.

## Slide 8
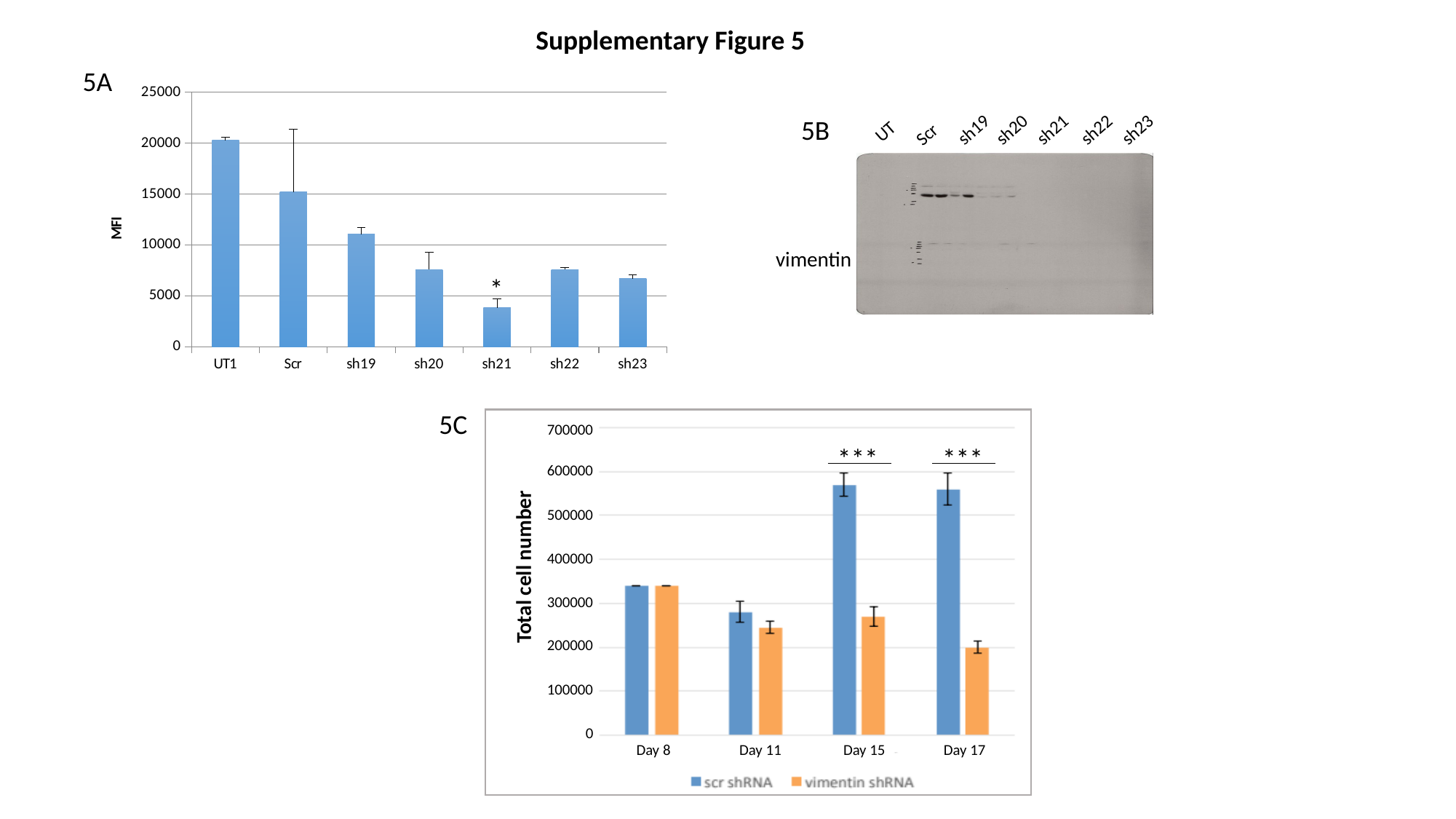

Supplementary Figure 5
### Chart
| Category | Vimentin |
|---|---|
| UT1 | 20289.0 |
| Scr | 15208.0 |
| sh19 | 11030.0 |
| sh20 | 7586.5 |
| sh21 | 3839.5 |
| sh22 | 7588.0 |
| sh23 | 6704.0 |5A
5B
sh19
sh20
sh21
sh22
sh23
UT
Scr
vimentin
*
5C
700000
***
***
600000
500000
400000
Total cell number
300000
200000
100000
0
Day 8 Day 11 Day 15 Day 17

## Slide 9
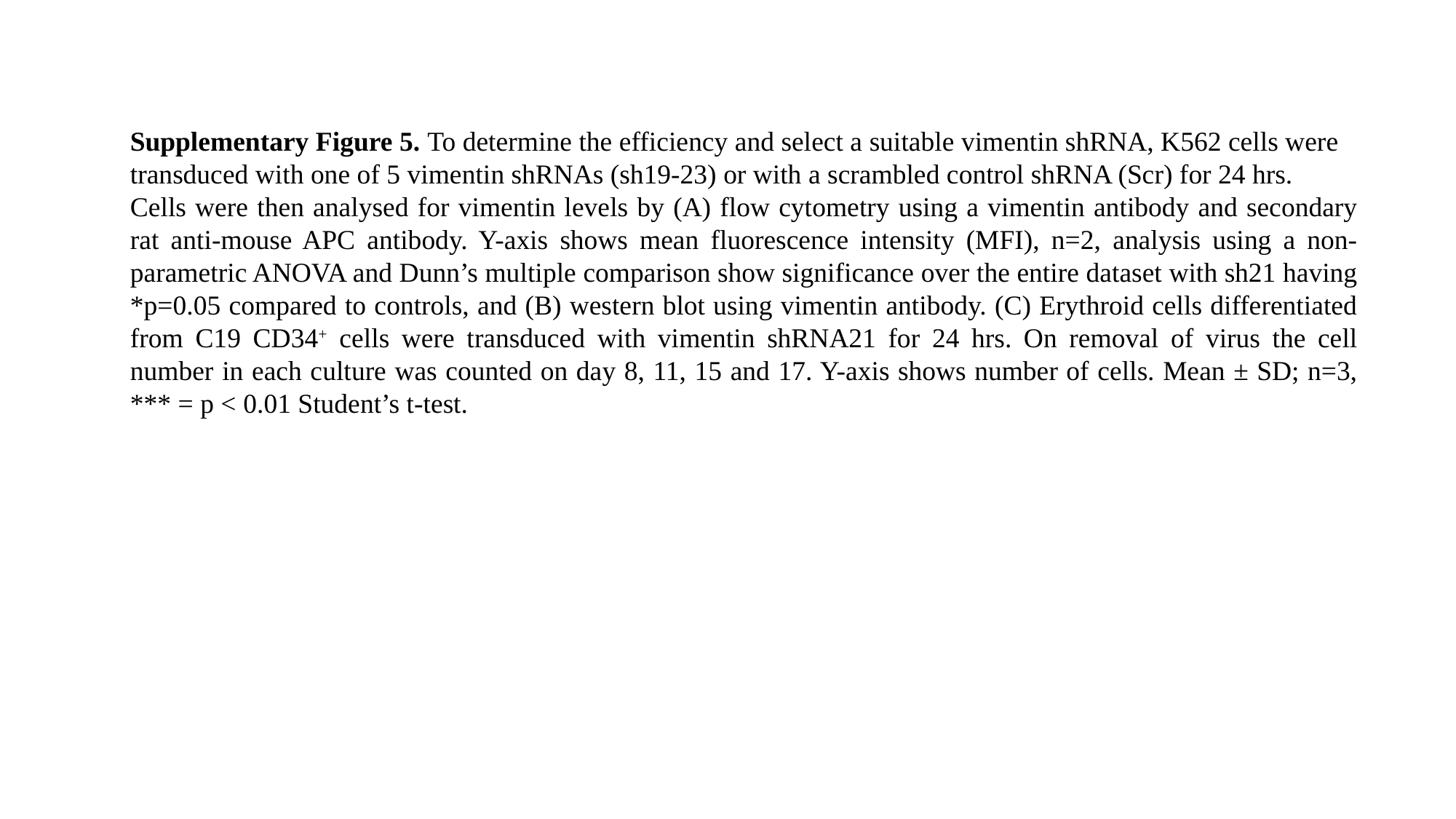

Supplementary Figure 5. To determine the efficiency and select a suitable vimentin shRNA, K562 cells were
transduced with one of 5 vimentin shRNAs (sh19-23) or with a scrambled control shRNA (Scr) for 24 hrs.
Cells were then analysed for vimentin levels by (A) flow cytometry using a vimentin antibody and secondary rat anti-mouse APC antibody. Y-axis shows mean fluorescence intensity (MFI), n=2, analysis using a non-parametric ANOVA and Dunn’s multiple comparison show significance over the entire dataset with sh21 having *p=0.05 compared to controls, and (B) western blot using vimentin antibody. (C) Erythroid cells differentiated from C19 CD34+ cells were transduced with vimentin shRNA21 for 24 hrs. On removal of virus the cell number in each culture was counted on day 8, 11, 15 and 17. Y-axis shows number of cells. Mean ± SD; n=3, *** = p < 0.01 Student’s t-test.

## Slide 10
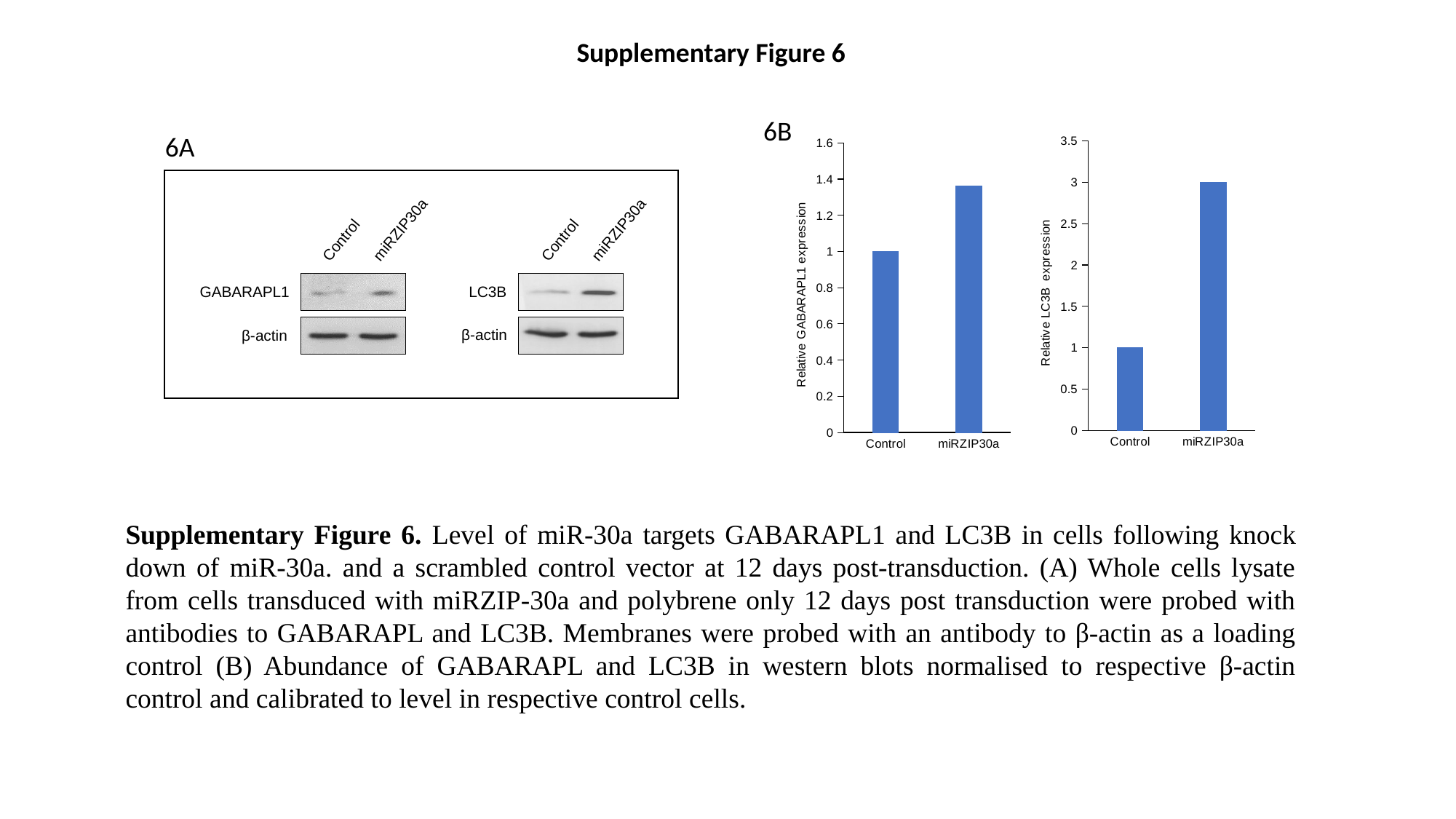

Supplementary Figure 6
6B
6A
### Chart
| Category | LC3B |
|---|---|
| Control | 1.0 |
| miRZIP30a | 2.994630842650403 |
### Chart
| Category | GABARAPL1 expression |
|---|---|
| Control | 1.0 |
| miRZIP30a | 1.3614908323017043 |
miRZIP30a
Control
GABARAPL1
β-actin
miRZIP30a
Control
LC3B
β-actin
Supplementary Figure 6. Level of miR-30a targets GABARAPL1 and LC3B in cells following knock down of miR-30a. and a scrambled control vector at 12 days post-transduction. (A) Whole cells lysate from cells transduced with miRZIP-30a and polybrene only 12 days post transduction were probed with antibodies to GABARAPL and LC3B. Membranes were probed with an antibody to β-actin as a loading control (B) Abundance of GABARAPL and LC3B in western blots normalised to respective β-actin control and calibrated to level in respective control cells.
